# Outcomes of Barotrauma in Critically Ill COVID-19 Patients With Severe Pneumonia

**DOI:** 10.1177/08850666211023360

**Published:** 2021-06-21

**Authors:** Victor P. Gazivoda, Mudathir Ibrahim, Aaron Kangas-Dick, Arony Sun, Michael Silver, Ory Wiesel

**Affiliations:** 1 Department of Surgery, 2042 Maimonides Medical Center, Brooklyn, NY, USA; 2 Division of Biostatistics, 2042 Maimonides Medical Center, Brooklyn, NY, USA; 3 Division of Thoracic Surgery, 2042 Maimonides Medical Center, Brooklyn, NY, USA

**Keywords:** COVID-19, coronavirus, barotrauma, pneumomediastinum, pneumothorax

## Abstract

**Background:**

Pneumomediastinum and pneumothorax are complications which may be associated with barotrauma in mechanically ventilated patients. The current literature demonstrates unclear outcomes regarding barotrauma in critically ill patients with severe COVID-19. The purpose of this study was to examine the incidence of barotrauma in patients with severe COVID-19 pneumonia and its influence on survival.

**Study Design and Methods:**

A retrospective cohort study was performed from March 18, 2020 to May 5, 2020, with follow-up through June 18, 2020, encompassing critically ill intubated patients admitted for COVID-19 pneumonia at an academic tertiary care hospital in Brooklyn, New York. Critically ill patients with pneumomediastinum, pneumothorax, or both (n = 75) were compared to those without evidence of barotrauma (n = 206). Clinical characteristics and short-term patient outcomes were analyzed.

**Results:**

Barotrauma occurred in 75/281 (26.7%) of included patients. On multivariable analysis, factors associated with increased 30-day mortality were elevated age (HR 1.015 [95% CI 1.004-1.027], *P* = 0.006), barotrauma (1.417 [1.040-1.931], *P* = 0.027), and renal dysfunction (1.602 [1.055-2.432], *P* = 0.027). Protective factors were administration of remdesivir (0.479 [0.321-0.714], *P* < 0.001) and receipt of steroids (0.488 [0.370-0.643], *P* < 0.001).

**Conclusion:**

Barotrauma occurred at high rates in intubated critically ill patients with COVID-19 pneumonia and was found to be an independent risk factor for 30-day mortality.

## Introduction

The Coronavirus Disease 2019 (COVID-19) pandemic resulted in a rapid increase in the number of patients requiring mechanical ventilation and intubation. Recent series suggest that during the initial wave of the pandemic, approximately 25% of patients required intubation.^1,2^ Our institution, a tertiary care academic medical center in Brooklyn, New York, was situated at the epicenter the first wave of the COVID-19 pandemic in New York City during early 2020.

SARS-COV-2, the virus associated with COVID-19, causes pathophysiologic changes in patients most consistent with the complex entity known as Adult Respiratory Distress Syndrome (ARDS). Traditional management strategies for patients with ARDS involve higher positive end-expiratory pressure (PEEP) ventilator settings to prevent repetitive alveolar collapse.^3^ Unfortunately, high PEEP values can be associated with lung injury resulting in barotrauma complications of pneumomediastinum (PM), pneumothorax (PTX), or a combination of both, although this relationship has not been conclusively proven.^3–7^ The uncertainty of this association arises as the use of low and high PEEP protocols in trials have not demonstrated a difference in barotrauma occurrence.^3,4,6,7^ The reported incidence of barotrauma in patients with COVID-19 is between 1% to 40%, and there is uncertainty regarding its influence on mortality.^8–17^

We aimed to characterize the incidence of barotrauma in critically ill mechanically ventilated patients with severe COVID-19 pneumonia and to examine the influence of these phenomena on survival. Our primary outcome was to investigate the influence of barotrauma on 30-day mortality in patients with COVID-19. Secondary outcomes included morbidity and disposition.

## Methods

### Study Design

An IRB-approved (Maimonides Institutional Review Board, approval # 2020-04-13) retrospective study encompassing data from March 18, 2020 to May 5, 2020 with follow-up through June 18, 2020, was undertaken at our medical center located in one of the epicenters of the COVID-19 pandemic in New York City. This period represented the initial surge of COVID positive patients at our institution which ranged from March to May 2020. All critically ill intubated patients admitted with a diagnosis of SARS-COV-2 as established by RT-PCR testing were included in the study. Patients who were not intubated and those who did not have a SARS-COV-2 positive RT-PCR were excluded. Barotrauma was defined as having pneumomediastinum, pneumothorax, or both. The diagnosis of barotrauma was established by searching the medical record for any of the above terms and confirmed by independent review of chest radiograph images. A consecutive series of 281 patients were selected based on these criteria, of which 75 patients had the diagnosis of barotrauma and were further stratified by PM, PTX, or both PM and PTX. Thirty-day outcomes including mortality and disposition were collected and analyzed.

### Statistical Analysis

All numeric variables are summarized with median (IQR) and were compared using the Kruskal-Wallis Test (non-parametric version of ANOVA). All categorial variables are summarized with frequency and percentage and compared with the chi-square test. A proportional hazards cox regression analysis was performed using time from intubation to death. A multivariable model was constructed using forward selection of variables significant at the 0.05 level for inclusion. All analyses were performed using SAS (SAS Institute, Cary, NC).

## Results

### Cohort Demographics

A total of 281 patients were included in the analysis. The median age in the barotrauma group was 63.5 (50-72) years versus 69 (60-76) years in patients without barotrauma (*P* = 0.001). Patients in both groups were similar in other characteristics, with the exception of asthma, for which there was a higher prevalence in the barotrauma group compared to the non-barotrauma group (13.3% and 5.9%, *P* = 0.039). In both groups, diabetes mellitus (DM) and hypertension (HTN) were the most frequent comorbidities (Table 1).

**Table 1.** Patient Cohort Characteristics.^a^

		Group		
Variable		Barotrauma (n = 75)	Control (n = 206)	*P*-value
Age (median, IQR)		**63.5 (50-72)**	**69 (60-76)**	**0.001**
BMI (median, IQR)		29.6 (25.9-33.1)	29.3 (25.8-34.05)	0.998
Sex	Male	50 (66.67%)	142 (68.93%)	0.718
	Female	25 (33.33%)	64 (31.07%)	
Race	White	41 (55.41%)	125 (60.98%)	0.070
	Black	4 (5.41%)	17 (8.29%)	
	Hispanic	18 (24.32%)	22 (10.73%)	
	Asian	7 (9.46%)	27 (13.17%)	
	Other	4 (5.41%)	14 (6.83%)	
Medical comorbidity	Arrhythmia	4 (5.33%)	22 (10.73%)	0.168
	**Asthma**	**10 (13.33%)**	**12 (5.85%)**	**0.039**
	Coronary artery disease	11 (14.67%)	37 (18.05%)	0.506
	COPD	2 (2.67%)	15 (7.32%)	0.149
	Cardiac surgery	4 (5.33%)	8 (3.9%)	0.601
	Diabetes mellitus	38 (50.57%)	109 (53.17%)	0.710
	Hyperlipidemia	29 (38.67%)	73 (35.61%)	0.638
	Hypertension	40 (52.00%)	129 (62.93%)	0.098
	History of renal failure	4 (5.33%)	18 (8.74%)	0.347

^a^ Bold text denotes statistically significant values.

### Novel COVID-19 Therapies

In light of the pandemic, many patients received novel therapies for COVID-19 pneumonia, with the most common being hydroxychloroquine (92.00% vs 88.83%), azithromycin (90.7% vs 88.4%), and ceftriaxone (82.7% vs 82.5%). No statistically significant difference was found between the barotrauma group and non-barotrauma group regarding administration of remdesivir (21.3% vs 16.0% respectively, *P* = 0.299) and steroids (68.0% vs 55.3% respectively, *P* = 0.057). The only therapy in which there was a statistically significant difference in administration was anti-interleuken-6 therapy (32.0% vs 19.4% respectively, *P* = 0.0261) (Table 2).

**Table 2.** Novel COVID-19 Treatments.^a^

Variable	Barotrauma (n = 75)	Control (n = 206)	*P*-value
Hydroxychloroquine	69 (92.00%)	183 (88.83%)	0.441
Azithromycin	68 (90.67%)	182 (88.35%)	0.5834
Ceftraxione	62 (82.67%)	170 (82.52%)	0.978
Remdesivir	16 (21.33%)	33 (16.02%)	0.299
Steroids	51 (68.00%)	114 (55.34%)	0.057
Anti-IL-6 therapy	**24 (32.00%)**	**40 (19.42%)**	**0.026**
Convalescent Plasma	4 (5.33%)	18 (8.74%)	0.347

^a^ Bold text denotes statistically significant values.

### Patient Outcomes

**Figure 1.**
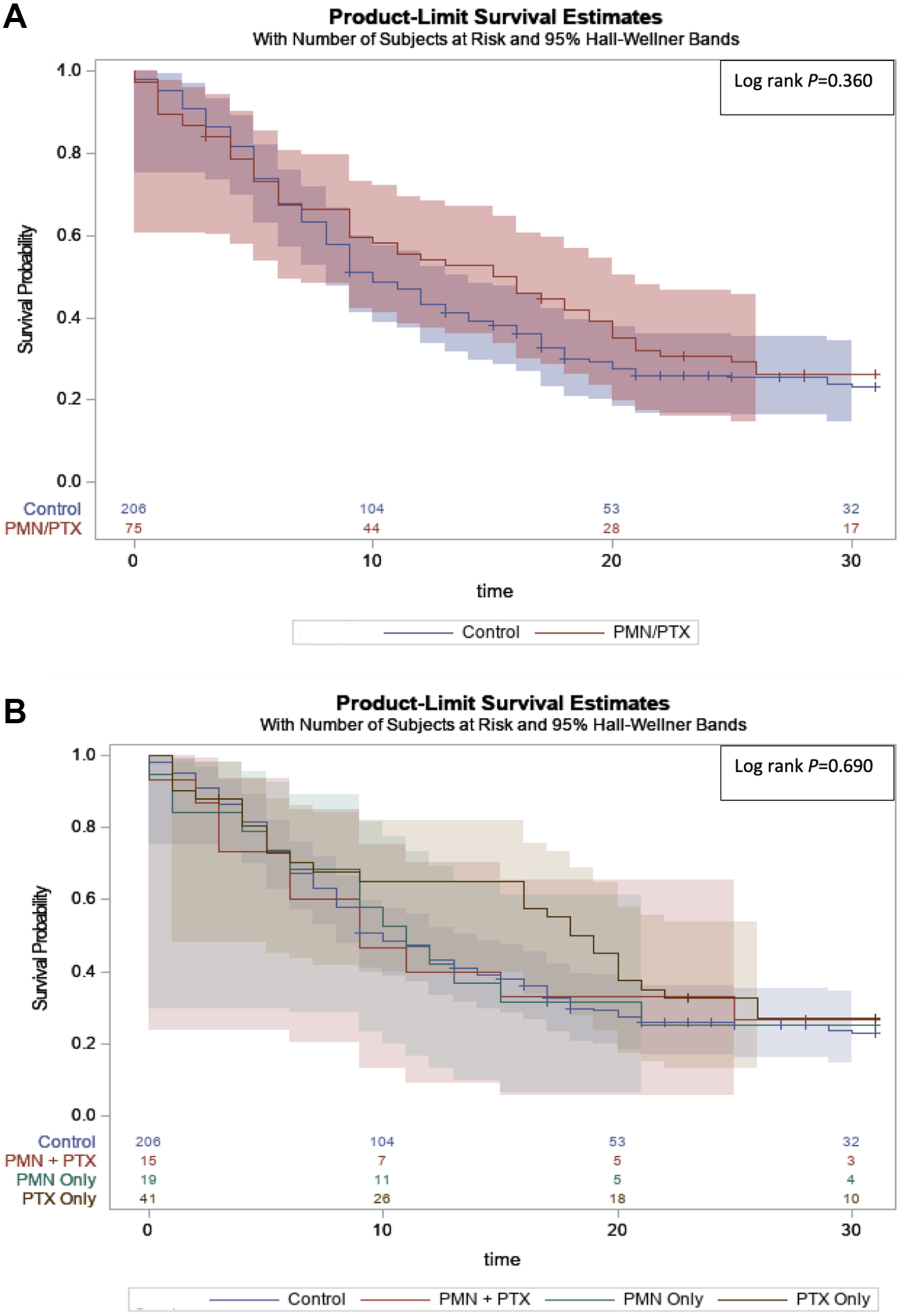
Kaplan Meier plot from survival regression analysis for patients by barotrauma incidence. A, Barotrauma Cohort. B, Stratified Barotrauma Cohort.

There were no statistically significant differences in overall outcomes between the barotrauma and non-barotrauma groups. The rate of 30-day mortality was 72.0% vs 74.8% between the two groups, (*P* = 0.641) (Table 3). On survival analysis, there was no statistically significant difference between both groups (Figure 1A). Analysis of the stratified barotrauma subgroups demonstrated similar median overall survival among the PM only, concomitant PM and PTX, and the non-barotrauma group, yet patients with PTX only appeared to have a trend toward a longer median overall survival (*P* = 0.690) (Figure 1B).

**Table 3.** 30-Day Patient Outcomes.^a^

Variable	Barotrauma (n = 75)	Control (n = 206)	*P*-value
Chest tube	**54 (72.00%)**	**1 (0.49%)**	**<0.001**
ICU LOS (days)	15 (5-22.5)	12 (6-19)	0.581
Total LOS (days)	17 (7-31)	14 (8-25)	0.270
Renal dysfunction during admission	53 (71.62%)	169 (82.04%)	0.058
Discharged	11 (14.67%)	39 (19.12%)	0.390
30-day mortality	54 (72.00%)	154 (74.76%)	0.641

Abbreviation: LOS, length of stay.

^a^ Bold text denotes statistically significant values.

On multivariable analysis, factors associated with 30-day mortality were elevated age (HR 1.015 [95% CI 1.004-1.027], *P* = 0.006), barotrauma (1.417 [1.040-1.931], *P* = 0.027), and renal dysfunction (1.602 [1.055-2.432], *P* = 0.027). Protective factors were administration of remdesivir (0.479 [0.321-0.714], *P* < 0.001) and receipt of steroids (0.488 [0.370-0.643], *P* < 0.001) (Table 4).

**Table 4.** Multivariable Cox Regression Analysis for Overall Survival at 30 Days.^a^

Variable		HR	95% CI	*P*-value
Barotrauma		**1.417**	**1.040-1.931**	**0.027**
Age		**1.015**	**1.004-1.027**	**0.006**
Sex	F	0.770	0.573-1.036	0.084
Remdesivir		**0.479**	**0.321-0.714**	**<0.001**
Renal Dysfunction		**1.602**	**1.055-2.432**	**0.027**
Steroids		**0.488**	**0.370-0.643**	**<0.001**

Abbreviations: HR, hazard ratio; PM, pneumomediastinum; PTX, pneumothorax.

^a^ Bold text denotes statistically significant values.

The stratified data of the barotrauma subgroups are included in Supplemental Tables 1, 2, and 3.

## Discussion

Our study represents one of the largest cohorts of critically ill mechanically ventilated COVID-19 patients who experienced barotrauma. We have demonstrated that critically ill intubated patients with COVID-19 have a high incidence of barotrauma complications (PM, PTX, or both) with an incidence of 26.7% (75/281). On multivariable analysis, factors associated with increased 30-day mortality were elevated age (with increased risk of 1.5% per year), barotrauma, and renal dysfunction. Protective factors for 30-day mortality were administration of remdesivir and receipt of steroids.

### Barotrauma in Mechanically Ventilated COVID-19 Patients

Barotrauma is the physical damage to body tissues caused by a difference in pressure between a gas space inside or in contact with the body surroundings.^18^ Pulmonary barotrauma is presumed to develop from rupture of hyperinflated alveoli causing air to leak into the surrounding tissues and spaces clinically manifested by interstitial emphysema, pneumothorax, pneumomediastinum, pneumoperitoneum, or subcutaneous emphysema.^18,19^ Patients with severe underlying lung disease such as ARDS, aspiration pneumonia, and pre-existing chronic obstructive lung disease have demonstrated increased risk of barotrauma.^18,19^

SARS-COV-2 is believed to bind ACE2 and subsequently Toll-like receptors on pneumocytes activating the host’s immune system and inducing the recruitment of inflammatory cells with subsequent production of pro-inflammatory cytokines and chemokines.^20^ This phenomenon results in interstitial and alveolar edema leading to ARDS. Dictated by the widely accepted ARDSnet protocol, patients with ARDS are managed with higher PEEP ventilator settings to prevent repetitive alveolar collapse.^3^ Such management can lead to high peak pressures and increased risk of barotrauma resulting in PM and PTX, although this relationship has not been conclusively proven_._^3–7^ The uncertainty of this association arises as the use of low and high PEEP protocols in trials have not demonstrated a difference in barotrauma occurrence.^3,4,6,7^Similarly, a recent publication by Kahn et al demonstrated no difference in tidal volume or average/peak airway pressures in patients with COVID-19 receiving invasive mechanical ventilation between those with barotrauma and those without barotrauma.^21^ While the underlying pathological mechanisms of PM or PTX in COVID-19 patients has yet to be fully elucidated, a widely postulated mechanism is that the severe and prolonged inflammation caused by the SARS-COV-2 virus leads to a widespread destruction of alveoli and airspace, increasing the likelihood of rupture with slight pressure changes from Valsalva maneuvers or mechanical ventilation.^22,23^ These mechanisms may explain the possible increased incidence of barotrauma complications associated with mechanically ventilated COVID-19 patients.

Increased barotrauma rates were previously reported during prior coronavirus epidemics; 12%-34% during the SARS-COV-1 pandemic (2002-2004) and 30% with the Middle East Respiratory syndrome coronavirus outbreak in 2014.^24–26^ The reported incidence of barotrauma in patients with COVID-19 varies in the literature. While earlier series, which included both intubated and non-intubated patients with COVID-19, reported a lower incidence of PTX,^11,12^ more recent studies of mechanically ventilated patients with COVID-19 have demonstrated increased incidence of barotrauma of 10%-40%,^8,9,13–17^ which is similar to our finding of an incidence of 26.7%. These reports suggest that barotrauma incidence during severe viral pneumonia is higher compared to the lower incidence reported in mechanically ventilated typical ARDS patients, which is primarily under 10%.^3,4,6,7,19^ This evidence further supports the theory that patients with COVID-19 have an increased risk of barotrauma related complications.

### Outcomes

Fifty-six (71.8%) patients with barotrauma were managed with tube thoracostomy. Of these patients, the majority had chest tubes placed for barotrauma induced PTX. The management of barotrauma in patients with COVID-19 can be clinically and technically challenging because of safety concerns and possible risk of virus dissemination.^27^ It remains unclear how chest tube management affects the overall outcome in this population. Though tube thoracostomy did not seem to change survival in our patient cohort, chest tube drainage remains the first line management for patients with pneumothorax related barotrauma and should be considered for drainage when clinically indicated.

As seen in previous studies, mortality rates were elevated during the COVID-19 pandemic.^1,2,8,28^ Most studies describing barotrauma and its influence on mortality in patients with COVID-19 are case reports and small series with conflicting results.^8–17^ Of note, the largest series to date presents 89 of 601 mechanically ventilated COVID-19 patients with barotrauma and reported no difference in the rate of mortality between COVID-19 patients with and without barotrauma. The authors further reported that barotrauma is an independent risk factor for death in their cohort.^8^ Our study validates and adds to these findings. We also found no difference in 30-day mortality rates between the barotrauma and non-barotrauma groups; however, on multivariable analysis, barotrauma was associated with 30-day mortality. Although this relationship initially appears difficult to understand, it could be explained by the barotrauma group in our study being younger and having a higher percentage of patients receiving steroids, which once controlled for on multivariable analysis, elucidated the association between barotrauma and mortality. This is consistent with our multivariable analysis demonstrating increased age to be a predictor of mortality and steroids being protective. Specifically, using age as a continuous variable, we found that for every 1-year increase in age, there was a 1.5% increased risk in mortality. Increased age has been consistently shown to be risk factors for severe disease and mortality in patients with COVID-19.^11,12,28–30^ In addition, glucocorticoids have been demonstrated to improve mortality in critically ill patients with COVID-19, with the greatest benefit being in patients who were receiving mechanical ventilation at the time of randomization.^31,32^ Furthermore, our analysis demonstrated renal dysfunction during admission to be predictive of increased mortality and administration of remdesivir to be protective. Supportive of these findings, several larger retrospective studies and meta-analyzes have demonstrated that renal dysfunction is a common serious complication of COVID-19, with older age and severe infection being independent risk factors for in-hospital death.^33–36^ In addition, the benefits of remdesivir for patients with COVID-19 have been previously described in the literature and continue to be investigated.^37–40^

Although further studies are required, we believe that most barotrauma in critically ill patients with COVID-19 can be managed successfully and safely using standard practice. More so, considering the high mortality rates compared to other patient populations with barotrauma, the likely determinant of the final clinical outcome in this population is the underlying pathophysiology and clinical course of the COVID-19 infection. As we have learned from cumulative research and data published since the initial wave of COVID-19, novel treatments such as corticosteroids and remdesivir have demonstrated improvement in these outcomes.

### Limitations

There are several important limitations to our study. First, we are reporting retrospective data from a single institution during the initial months of the COVID-19 pandemic, which may not be generalizable to all populations and times of the pandemic. Second, given the significant resource constraints associated with the pandemic, data documentation was at times incomplete; therefore, certain variables such as ventilatory settings and smoking status were not attainable in our chart review. Although our institution followed national and international guidelines for treatment of critically ill COVID-19 patients and treated intubated COVID-19 patients within the “lung protective” directives of the ARDSnet protocol as previously described,^41^ it should be noted that given the clinical situation, there was some heterogeneity in ventilator management despite guidelines. Additionally, although there were no documented procedural complications on review of the electronic medical record, other causes for barotrauma (such as iatrogenic procedural causes) cannot be completely ruled out. Furthermore, our search criteria for complications of barotrauma were defined as pneumothorax, pneumomediastinum, or both. The definition of barotrauma can also include subcutaneous emphysema. Although certain patients in the barotrauma cohort had subcutaneous emphysema, all patients with subcutaneous emphysema may not have been included in the cohort. Additionally, patients with COVID pneumonia were not stratified based on extent of disease and a diagnosis of ARDS as defined by the Berlin Criteria, which could have further contributed to these patients’ outcomes. Lastly, though the effects of novel COVID-19 treatments were not known, and the availability and dissemination of these regimens were implemented at different time periods throughout the study, our facility was able to utilize these treatments for critically ill patients. The benefits of some of these treatments have now demonstrated promising results, while others were not found to be effective based on accumulating evidence and studies similar to ours.

## Interpretation

Our study represents one of the largest cohorts of critically ill intubated patients with COVID-19 severe pneumonia who experienced barotrauma. Barotrauma occurred at a high incidence and appears to be associated with increased 30-day mortality. Receipt of corticosteroids and remdesivir were associated with improved outcomes.

## Supplemental Material

Supplemental Material, sj-pdf-1-jic-10.1177_08850666211023360 - Outcomes of Barotrauma in Critically Ill COVID-19 Patients With Severe Pneumonia Click here for additional data file. Supplemental Material, sj-pdf-1-jic-10.1177_08850666211023360 for Outcomes of Barotrauma in Critically Ill COVID-19 Patients With Severe Pneumonia by Victor P. Gazivoda, Mudathir Ibrahim, Aaron Kangas-Dick, Arony Sun, Michael Silver and Ory Wiesel in Journal of Intensive Care Medicine
